# Kinetics and Phenotype of the CD4 T Cell Response to Influenza Virus Infections

**DOI:** 10.3389/fimmu.2019.02351

**Published:** 2019-10-02

**Authors:** Emma E. Hornick, Zeb R. Zacharias, Kevin L. Legge

**Affiliations:** ^1^Interdisciplinary Immunology Graduate Program, Department of Pathology, University of Iowa, Iowa City, IA, United States; ^2^Department of Microbiology and Immunology, University of Iowa, Iowa City, IA, United States

**Keywords:** influenza virus, CD4 T cells, adaptive immune response, pulmonary immunity, T helper subsets

## Abstract

Influenza A virus (IAV) is a leading cause of respiratory infections, with increased risk of severe illness and death in the very young, aged, and immunocompromised individuals. In both mice and humans, IAV-specific T cell responses are protective during primary as well as homologous and heterologous challenge infections. Many mouse studies have focused on CD4 T cells specific for a single, known model or IAV antigen. However, studies have demonstrated that the IAV-specific CD4 T cell response comprises many epitopes spread across multiple viral proteins. Therefore, herein we track the antigen-experienced CD4 T cell response using the surrogate markers CD49d and CD11a. This novel surrogate marker method allows us to characterize the full IAV-specific CD4 T cell response without the potential bias that could occur when examining an individual Ag-specificity. Our findings demonstrate that the immunodominant I-A^b^-binding NP_311−325_ epitope often used in studies of IAV-specific CD4 T cells represents only about 5% of the total IAV-specific CD4 T cell response. Further, we find that the kinetics of the full pulmonary CD4 T cell response is similar to that of NP_311_-specific T cells and that the full CD4 T cell response in the lungs is predominantly composed of cells expressing the Th1 transcription factor T-bet, with smaller but significant portions of the response expressing the Treg and Tfh associated transcription factors Foxp3 and Bcl-6, respectively. Interestingly, although Th1 cells are the most abundant Th subset in the lungs of both BALB/c and C57Bl/6 mice following IAV, the relative abundance of Treg and Tfh is reversed in the different mouse strains. In BALB/c mice, Foxp3^+^ cells are more abundant than Bcl6^+^ cells, whereas in C57Bl/6 mice, there are more Bcl6^+^ cells. As a whole, these data highlight the diversity of the endogenous CD4 T cell response to a primary IAV infection, providing an important context for past and future studies of the IAV-specific CD4 T cell response.

## Introduction

Influenza A virus (IAV) is a major cause of respiratory infection, leading to ~200,000 hospitalizations and 36,000 deaths in the United States each year (1). Annual vaccination effectively induces antibody responses that can protect the host against infection with homologous viruses; more recent findings have also indicated that IAV-specific T cells generated following vaccination or a prior infection correlate with improved outcomes following subsequent IAV infection (2–4). Further recent analysis of murine and human lungs has demonstrated the presence of tissue resident IAV-specific T cells that enhance protection upon subsequent IAV exposures (5–7). Thus, the protective capacity of T cells during IAV infection highlights the importance of having a thorough understanding of the requirements for and characteristics of these responses.

During IAV infection, CD4 T cells provide help to both the B cell and CD8 T cell responses, produce pro-inflammatory cytokines and were recently shown to directly kill IAV-infected cells (8–11). Within the IAV-specific CD4 T cell response, IFNγ-producing Th1 cells seem to predominate, and important roles for both regulatory T cells and T follicular helper cells (Tfh) have also been established (12–16). To date, much of our understanding of the role of individual IAV-specific CD4 T helper subsets in the immune response to influenza virus comes from studies using adoptive transfer of TCR transgenic IAV-specific CD4 T cells or after *in vitro* activation and differentiation of IAV-specific T cells using a single or limited number of known IAV epitopes (8, 10, 14, 16–18). However, it is now appreciated that the CD4 T cell response to IAV is made up of 10–100 s of epitope specificities (19–22). Thus, it currently remains unclear how representative the findings from studies utilizing T cells specific for a single or limited number of epitopes are of the full diverse endogenous CD4 T cell response to influenza virus.

In this study, we use a novel method of tracking antigen-experienced CD4 T cells using the surrogate markers CD49d and CD11a, allowing us to quantify the full IAV-specific CD4 T cell response without prior knowledge of the precise antigen specificity of each individual T cell within the response (23–27). We find that while the kinetics of the full response are similar to those observed when only T cells of a known epitope specificity are tracked, the complete CD4 T cell response within the lungs after an IAV infection is several times larger than the response specific for the immunodominant I-A^b^-binding NP_311−325_ epitope often used in studies of the IAV-specific CD4 T cell response. We demonstrate that the endogenous CD4 T cell response found in the lungs during IAV infections is predominantly composed of T-bet^+^ cells, with smaller but significant populations of Foxp3^+^ or Bcl-6^+^ cells. Interestingly, the antigen-experienced CD4 T cell response in BALB/c mice shows a similar kinetics and T-bet dominance to that observed in C57Bl/6 mice. However, differences in the ratio of Foxp3^+^ to Bcl-6^+^ cells between C57Bl/6 and BALB/c mice were observed. As a whole, these data indicate that the endogenous CD4 T cell response to a primary influenza virus infection is quite large and skewed toward T-bet^+^, FoxP3^+^, or Bcl-6^+^ Th subsets.

## Materials and Methods

### IAV Infection of Mice

Wild type female BALB/c and C57BL/6 mice were bred, housed, and maintained in the University of Iowa (Iowa City, IA) animal care facilities. All procedures were performed on matched mice, were approved by the Institutional Animal Care and Use Committee of the University of Iowa and comply with the NIH Guide for Care and Use of Laboratory Animals. Mice were randomly assigned into groups for each experiment. Age- and weight-matched groups of mice were lightly anesthetized by isoflurane inhalation and infected intranasally with a 0.1, 0.05, or 0.01 LD_50_ dose of mouse-adapted A/Puerto Rico/8/1934 (H1N1) (PR8) in 50 μL of Iscove's DMEM (Gibco). Virus was grown in the allantoic fluid of 10-day-old embryonated hen eggs for 2 days at 37°C. Allantoic fluid was harvested and stored at −80°C until use as previously described (28).

### Preparation of Cells

Lungs were harvested into 10 mL Iscove's DMEM, mashed through a wire mesh and filtered through a nylon mesh to obtain a single cell suspension. In some experiments, lungs were minced and digested in Iscove's media containing 1 mg/mL collagenase (Sigma) and 0.02 mg/mL DNAse (Sigma) for 15 min at 37°C prior to mashing. Live cells were quantified using trypan blue exclusion and a hemocytometer.

### Flow Cytometry

Antibodies were purchased from BD Biosciences (San Diego, CA), eBioscience (San Diego, CA), Tonbo biosciences (San Diego, CA), and BioLegend (San Diego, CA). The following monoclonal antibodies were used for these studies: anti-CD4 (GK1.5 and RM4-5, conjugated to FITC, PE, PerCP-Cy5.5, APC, PE-Cy7, BV421, eFluor450, and BV786), anti-CD8α (53-6.7 conjugated to FITC, PE, PerCP-Cy5.5, APC, APC-Cy7, PE-Cy7, BV421, eFluor450, AlexaFluor 700, and BV786), anti-CD90.2 (30-H12, conjugated to FITC, APC, APC-Cy7, PE-Cy7, BV421, BV786 and AlexaFluor 700), anti-CD11a (M17/4, conjugated to FITC, PE and eFluor450), anti-CD49d (R1-2, conjugated to FITC and AlexaFluor 647), anti-IFNγ (XMG1.2, conjugated to APC), anti-IL-2 (JES65H4, conjugated to PE and PE-Cy7), anti-IL-13 (eBio13A, conjugated to PE), anti-IL-17A (TC11-18H10.1, conjugated to PE), anti-Bcl-6 (K112.91 and Bcl-DWN, conjugated to PE and APC), anti-Foxp3 (FJK16s, conjugated to PE and eFluor450), anti-GATA-3 (TWAJ, conjugated to PE and APC), anti-RORγt (B2D, conjugated to PE and APC), anti-T-bet (eBio410, conjugated to PE-Cy7 and APC). I-A^b^: NP_311−325_ tetramers and CLIP-containing tetramer controls conjugated to PE and APC were obtained from the National Institute of Allergy and Infectious Disease-funded NIH Tetramer Core Facility at Emory University.

Cells were blocked with 2% rat serum in FACS buffer (sterile PBS, 2% heat-inactivated fetal calf serum, 0.02% Sodium Azide) for 15 min on ice, stained with the indicated antibodies for 30 min, and fixed with BD FACS Lysing Solution (BD Biosciences, San Diego, CA). For intracellular staining, surface staining was performed as described, followed by fixation, permeabilization and staining using the Foxp3 transcription factor staining kit per manufacturer's instructions (eBioscience, San Diego CA). For NP_311_-tetramer and CLIP-tetramer control staining, cells were stained in azide-free FACS buffer for 90 min at room temperature, followed by surface staining as above. Samples were acquired on a FACSCanto II or LSR II (BD Biosciences, San Diego, CA) using FACS Diva software and analyzed with FlowJo software (Treestar, Ashland, OR).

### Intravascular Stain to Determine Cellular Localization

Mice were administered 1 μg of BV421-conjugated rat anti-mouse CD45.2 (104) in 200 μL of PBS by retroorbital intravenous injection 3 min before euthanasia as previously described (29).

### MACS Purification of Splenic Stimulator DC and Overnight Peptide Pulse

CD11c^+^ splenocytes were enriched to 80–85% purity by positive selection (anti-CD11c MACS microbeads) following manufacturer instructions (Miltenyi Biotec, Auburn, CA). CD11c^+^ splenocytes were then incubated overnight at 37°C in complete medium with or without 10 μM NP_311−325_ peptide. After overnight incubation, cells were washed once and added to single cell suspensions of lung cells.

### Stimulation for Intracellular Cytokine Staining

Lung single cell suspensions in Iscove's complete medium (Iscove's DMEM, 2-mercaptoethanol, 10% heat-inactivated fetal calf serum, sodium pyruvate, penicillin and streptomycin) were incubated with splenic stimulator DC (3:2 ratio of DC to lung cells) that had been incubated with or without NP_311_ for 6 h in the presence of IL-2 (40 U/mL) and BFA (10 μg/mL) at 37°C.

### Statistical Analysis

Experiments were repeated at least twice unless noted otherwise. Comparisons between two groups were performed with a two-tailed student's *t*-test. Comparisons between more than two groups at different time points were analyzed using two-way ANOVA with Tukey's multiple comparison *post-hoc* test. A *P* ≤ 0.05 was considered significant.

## Results

**Figure 1:**
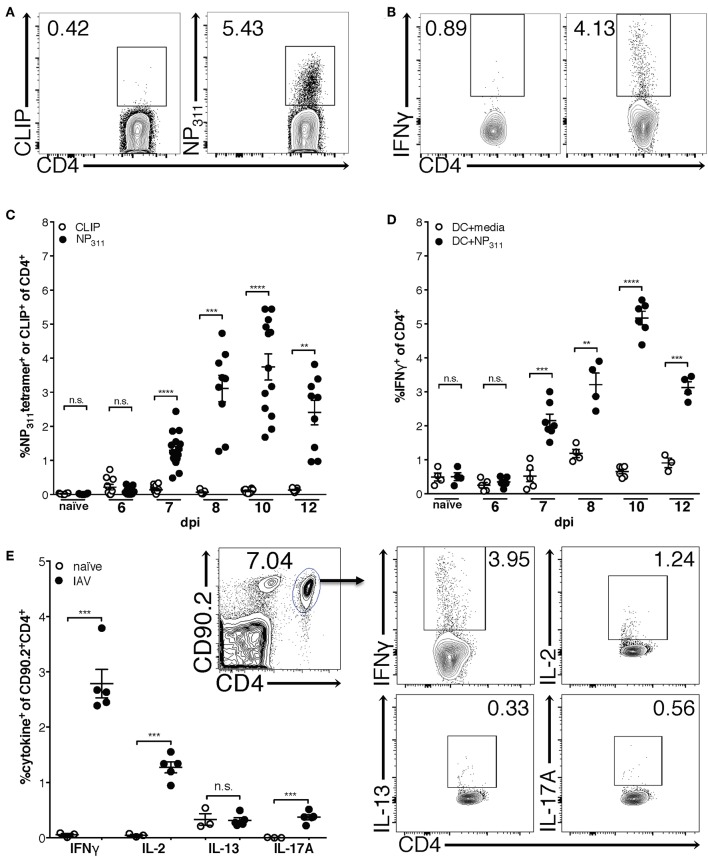
The frequency of NP_311_-specific CD4 T cells in the lungs peaks at 10 dpi. Mice were infected with a 0.05LD_50_ inoculum of IAV-PR8 and lungs were harvested at the indicated times post-infection. The frequency of NP_311_-specific CD4 T cells in the lung at each time point was determined by tetramer staining [representative flow plots (day 10 p.i., **A**) and quantification **(A,C)**] or intracellular staining for IFNγ following incubation with NP_311_-peptide pulsed splenic DC [representative flow plots (day 10 p.i., **B**) and quantification **(B,D)**]. CD4 T cells from the lungs on day 7 after infection with 0.05LD_50_ IAV-PR8 were analyzed for expression of IFNγ, IL-2, IL-13, IL-17A following incubation with NP_311_-peptide pulsed spleen DC **(E)**. Samples were analyzed by flow cytometry. Representative flow cytometry plots are from IAV infected mice. Each point represents one mouse and mean and SEM are shown. *n* = 3–15 mice per time point from 2 to 3 separate experiments. **p < 0.01, ***p < 0.001; ****p < 0.0001.

Previous studies using adoptively transferred CD4 T cells specific for a single IAV or model antigen epitope predict that the majority of the IAV-specific response is Th1-polarized and produces IFNγ (30). Therefore, in order to begin to clarify the kinetics of the endogenous CD4 T cell response to IAV, we quantified NP_311_-specific CD4 T cells [tetramer^+^ (Figure 1A) and IFNγ^+^ (Figure 1B)] during a primary IAV infection. NP_311_-specific cells are first significantly elevated above naïve levels at day 7 post-infection (p.i.), and peak at day 10 p.i. (Figures 1C,D). Further, the NP_311_-specific CD4 T cell response appears to be dominated by Th1 cells as the cytokine production by these cells was highlighted by IFNγ production with more limited IL-2 and IL-17 production (Figure 1E). We observed no increase in cells producing IL-13 in IAV infected vs. naive mice (Figure 1E).

**Figure 2:**
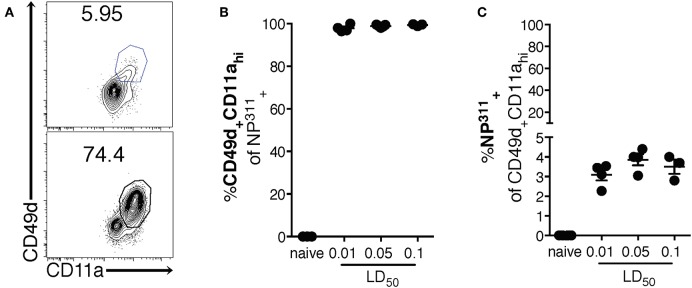
The majority of pulmonary NP_311_-specific CD4 T cells in the lungs are CD49d^+^CD11a^hi^ however NP_311_-specific CD4 T cells only account for a small fraction of the total IAV-specific CD4 T cell response. Mice were infected with a 0.1LD_50_, 0.05LD_50_, or 0.01LD_50_ dose of IAV-PR8 and the lungs were harvested and analyzed for NP_311_ specificity **(B,C)** and CD49d^+^CD11a^hi^ expression **(A–C)** at day 7 p.i. Shown are the representative plots of CD49d^+^CD11a^hi^ cells of CD4^+^ in naïve and 0.05LD_50_ IAV-infected mice **(A)**, the frequency of CD49d^+^CD11a^hi^ of CD4^+^NP_311_-tetramer^+^ cells **(B)**, or NP311-tetramer^+^ cells of CD4^+^CD49d^+^CD11a^hi^ gated cells **(C)**. Each point represents one mouse and mean and SEM are shown. Data are from one experiment, *n* = 3–4 mice per dose of infection.

Interestingly, although the NP_311_-specific CD4 T cell response was substantially larger than the response to other epitopes we tested (data not shown), it represents only ~4–5% of the total CD4 T cells in the lungs at the peak of the response (Figures 1C,D), highlighting the need for a method of identifying and tracking the full magnitude of the IAV-specific CD4 T cell response. Therefore, we addressed this need with a previously described strategy of identifying antigen-experienced CD4 T cells by co-expression of CD49d and CD11a (Figure 2A). This surrogate marker technique has proven useful in identifying antigen specific cells in several infections and species including those where epitopes have been poorly defined and in outbred populations where determination of the epitope specificity is difficult (23–27). As expected, IAV infection increases the percentage of CD4 T cells within the lungs that are CD49d^+^CD11a^hi^ vs. naïve mice (Figure 2A). Further 96–99% of NP_311_-specific CD4 T cells in the lungs, regardless of the infection dose, are CD49d^+^CD11a^hi^ at day 7 p.i. (Figure 2B). Consistent with our findings in Figure 1, the NP_311_-specific population only represents about 4% of the total antigen-experienced (CD49d^+^CD11a^hi^) CD4 T cells in the lungs (Figure 2C). These results not only confirm that NP_311_-specific cells make up a minority of the total IAV-specific CD4 T cell response, but also support the use of CD49d and CD11a to quantify and examine the full magnitude of the antigen-experienced CD4 T cell response within the lungs during IAV infections.

**Figure 3:**
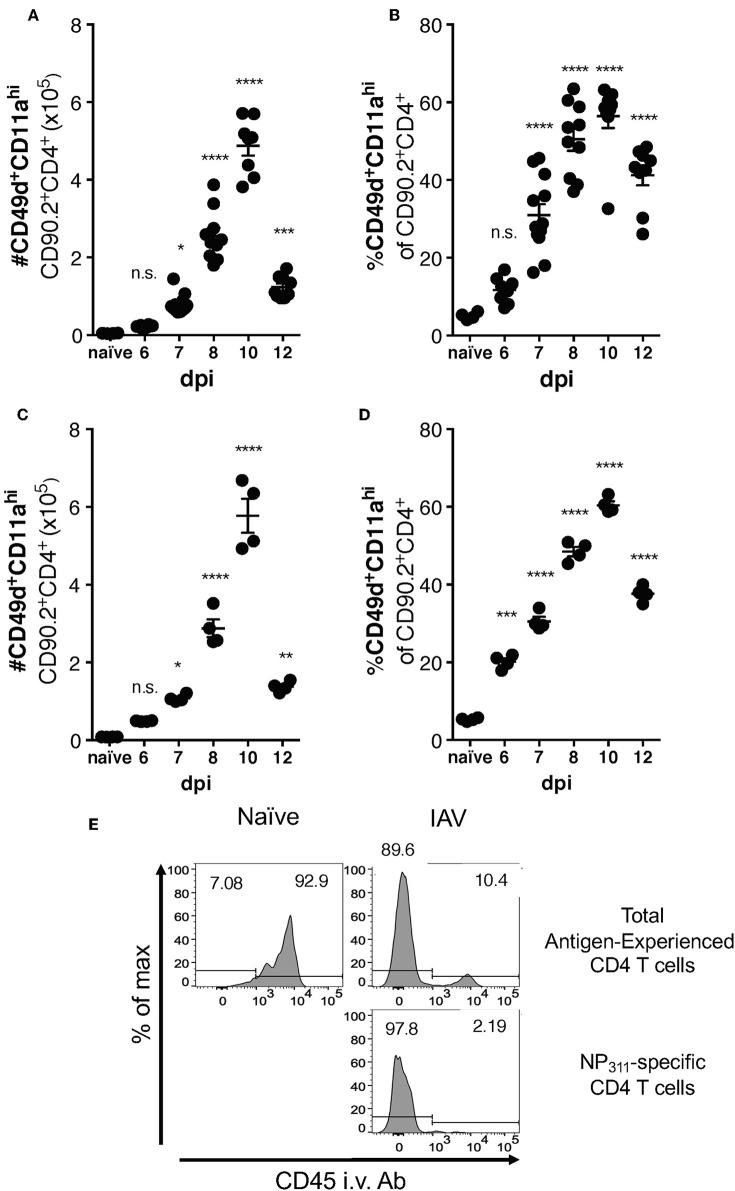
Accumulation of IAV-specific CD4 T cells in the lungs occurs with similar kinetics in C57Bl/6 and BALB/c mice. C57Bl/6 **(A,B)** and BALB/c **(C,D)** mice were infected with a 0.05LD_50_ dose of IAV and then the lungs were harvested and stained for surface expression of CD49, CD11a, CD90.2, and CD4 at indicated time points. A group of naïve mice was included as a control. Number **(A,C)** and frequency **(B,D)** of CD49d^+^CD11a^hi^ CD90.2^+^CD4^+^ T cells were quantified by flow cytometry. Each point represents one mouse and mean and SEM are shown. *n* = 4–10 mice, representative of at least three independent experiments for each mouse strain. *p < 0.05, **p < 0.01, ***p < 0.001; ****p < 0.0001, vs. naïve values. **(E)** C57Bl/6 mice were infected with a 0.1LD_50_ dose of IAV on day 10 p.i. the fraction of indicated T cells within the lungs or the circulation vs. measured via administration of anti-CD45-BV421 mAb i.v. 3 min prior to harvest.

Differences between the epitope specificities present in the IAV-specific CD4 T cell responses of C57Bl/6 and BALB/c mice have been documented, but a direct comparison of the magnitude of this response has not been performed. We therefore took advantage of the surrogate marker approach to address this gap in knowledge. In both mouse strains, the kinetics of the response was largely similar, with the number (Figures 3A,C) and frequency (Figures 3B,D) of CD49d^+^CD11a^hi^ CD4 T cells in the lungs peaking at day 10 p.i. The kinetics of accumulation of Ag-experienced CD4 T cells during the total endogenous response are consistent with the kinetics of accumulation reported in adoptive transfer models, and are also reflected in the endogenous NP_311_-specific response (Figure 1). Further as has been previously reported for IAV and after other pulmonary virus infections (24, 29, 31), the vast majority ~90% of the antigen-experienced CD4 T cells found within the lungs after IAV, but not naive mice, are found within the lung interstitium rather than the circulation/small capillary beds within the lungs (Figure 3E). As a whole, these data show that the magnitude and kinetics of the pulmonary CD4 T cell response during IAV infections is similar in BALB/c and C57Bl/6 mice.

Significant differences in the Th subset dominance and distribution of the CD4 T cell responses in BALB/c and C57Bl/6 mice has been observed during infections [e.g., Leishmania (18), etc.]. To our knowledge, a direct comparison of the Th response during IAV infection has not be undertaken, despite widespread use of both mouse strains. Furthermore, studies utilizing adoptive transfer of *in vitro* differentiated Th subsets have been utilized to determine the potential contributions of these subsets to IAV immunity. Therefore, to determine which Th subsets were present in the full endogenous CD4 T cell response, and whether there were differences in the Th subsets present in the lungs between these two mouse strains, we quantified IAV-specific Th1, Th2, Th17, Treg, and Tfh cells at several time points following IAV infection.

**Figure 4:**
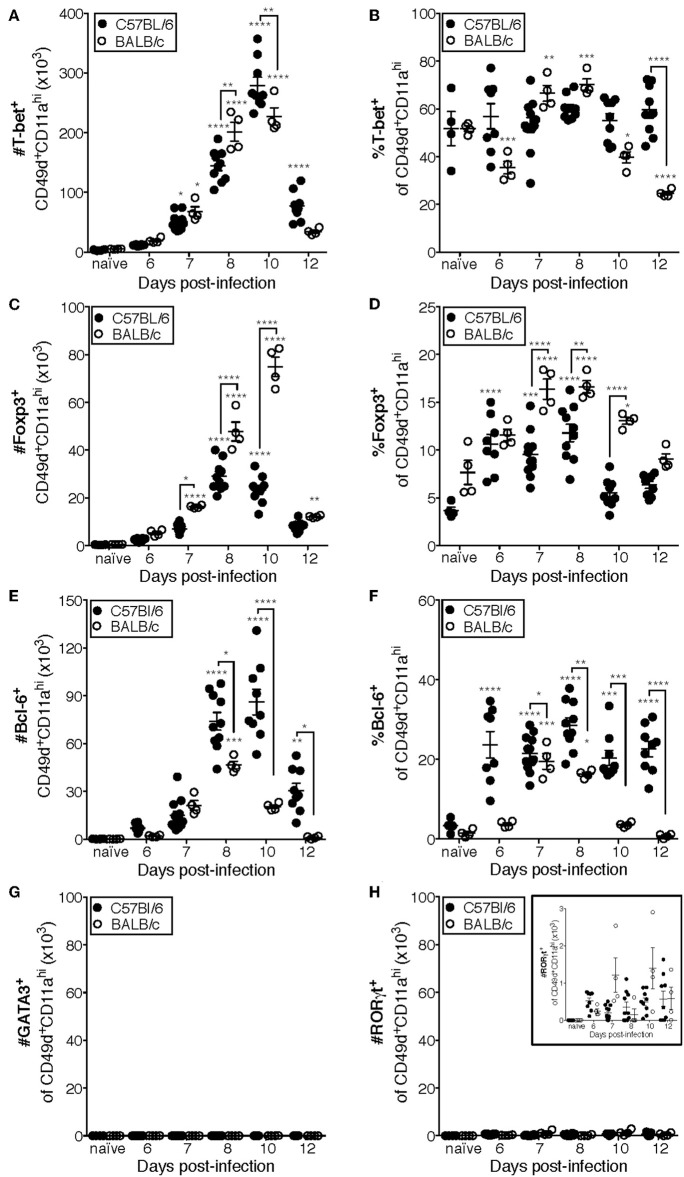
Most IAV-specific CD4 T cells accumulating in the lungs of C57Bl/6 and BALB/c mice following infection express T-bet, Bcl-6, or Foxp3. C57Bl/6 and BALB/c mice were infected with a 0.05LD_50_ inoculum of IAV-PR8. Lungs were subsequently harvested at the indicated time post-infection and the cells stained for surface expression of CD11a CD49d and CD4, fixed, permeabilized and then stained intracellularly for T-bet **(A,B)**, Bcl-6 **(C,D)**, Foxp3 **(E,F)**, GATA-3 **(G)**, or RORγt **(H)** expression. Cells were then analyzed by flow cytometry. The numbers and frequencies of T-bet^+^, Bcl-6^+^, Foxp3^+^, GATA-3^+^, or RORγt^+^ subsets of CD49d^+^CD11a^hi^CD4^+^ cells are shown. Each point represents one mouse and mean and SEM are shown. Data are representative of at least three independent experiments and *n* = 4–9 mice per time point. *p < 0.05; **p < 0.01; ***p < 0.001; ****p < 0.0001; asterisks directly above data points indicate significance relative to naïve values; asterisks above brackets indicate significance between bracketed groups.

Previous studies have indicated that the CD4 T cell response to IAV is predominantly composed of IFNγ-producing cells (10, 18). Consistent with those findings, the majority (~50–70%) of CD49d^+^CD11a^hi^ CD4 T cells are T-bet^+^ in the lungs of both C57Bl/6 and BALB/c mice at all measured time points (Figures 4A,B, Supplementary Data Sheet 1). These data are also consistent with Figure 1, in which the relative frequency of NP_311_-specific CD4 T cells producing IFNγ (Figure 1E) suggests that the majority of NP_311_-specific cells are capable of producing IFNγ, the signature cytokine of the Th1 subset.

T follicular helper (Tfh, Bcl-6^+^) cells provide help to the B cell response in a variety of contexts including IAV infection (13, 32, 33). In C57BL/6 mice, the number of Tfh present in the lungs after IAV is significantly elevated above naïve lungs at day 8 p.i. and peaks in number at day 10 p.i. (Figure 4C). Furthermore, the frequency of Tfh among the antigen-experienced (CD49d^+^CD11a^hi^) CD4 T cell pool is elevated from day seven until at least day 12 p.i. in the lungs of C57BL/6 mice (Figure 4D). In contrast, the number of Tfh in BALB/c mice is only significantly elevated above the number present in naïve lungs on day 8 p.i., which is also the peak of this response (Figure 4C). Similarly, the proportion of Tfh among the antigen-experienced (CD49d^+^CD11a^hi^) CD4 T cell pool in the lungs of BALB/c mice was only significantly elevated relative to naïve mice at days 7 and 8 p.i. (Figure 4D).

IAV-specific regulatory T cells (Treg, Foxp3^+^) have been shown to be important in dampening inflammatory signals and proliferation of IAV-specific CD4 and CD8 T cells during infection, thereby preventing excessive damage to host tissues (34, 35). The peak in the number of Treg cells in the lungs of C57BL/6 mice after IAV coincides with the peak in the number of total antigen-experienced CD4 T cells, at day 10 p.i. (Figure 4E), and the frequency of Treg cells among the antigen-experienced (CD49d^+^CD11a^hi^) CD4 T cell pool is significantly elevated above naïve at days 6 through 8 p.i. (Figure 4F). In the lungs of BALB/c mice, the number of Treg cells is significantly increased above naïve lungs from 7 to 12 days p.i., peaking day 10 p.i. (Figure 4E). Further, the frequency of pulmonary Treg cells among the antigen-experienced (CD49d^+^CD11a^hi^) CD4 T cell pool in the lungs of BALB/c mice is first significantly elevated above naïve lungs at day seven post-infection and peaks at days 7 and 8 post-infection (Figure 4F).

Th2 (GATA-3^+^) cells are detrimental during IAV infection, as manipulation of the host to increase the number of these cells resulted in substantially poorer outcomes (36, 37). Consistently, we did not detect a significant number of antigen-experienced CD4 T cells expressing GATA-3 in the lungs of either C57Bl/6 mice or BALB/c mice (Figure 4G). The role of Th17 cells during IAV infection remains largely unclear, as they have only been reported to constitute a large proportion of the IAV-specific CD4 T cell response in IL-10-deficient hosts (17). In agreement with this, while RORγt^+^ cells are present in the lungs (Figure 4H and insert) of both C57BL/6 and BALB/c mice, the number of these cells during IAV infection is never significantly elevated relative to naïve lungs (Figure 4H).

**Figure 5:**
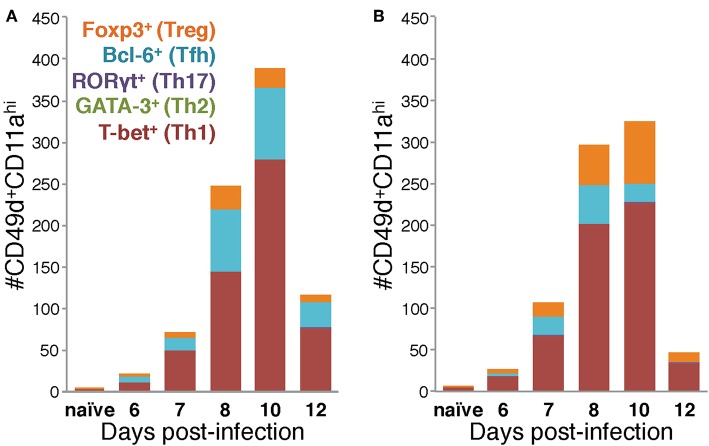
Differences in the Th subset composition of the IAV-specific CD4 T cell compartment in the lungs of BALB/c and C57Bl/6 mice during infection. Number of IAV-specific CD4 T cells corresponding to each of the indicated Th subsets in the lungs of IAV-infected C57Bl/6 **(A)** and BALB/c **(B)** mice based on their transcription factor expression.

Using the data in Figure 4 to directly compare the character and kinetics of the CD4 T cell subset response in the lungs after IAV infection between BALB/c and C57Bl/6 (Figures 4, 5) reveals important differences. At day 8 p.i., there are slightly (1.4x) but significantly more Th1 cells in the lungs of BALB/c mice compared to C57Bl/6 mice whereas at day 10 p.i., the number of Th1 cells in the lungs of C57Bl/6 mice overtakes the level observed in the lungs of BALB/c mice. The magnitude of the Tfh response is strikingly and significantly larger in C57BL/6 mice compared to BALB/c mice at days 8–12 p.i., both in number and frequency (Figures 4C,D). In contrast, the Treg response was significantly larger in the lungs of BALB/c mice than in C57Bl/6 mice at days 7–10 p.i., both in number and fraction of Tregs among the total CD4 T cell response (Figures 4E,F). These biases suggest that environmental factors in the BALB/c mouse may favor Treg vs. Tfh development while factors present in C57Bl/6 mice may instead favor Tfh responses.

Considered as a whole, the data in Figures 4, 5 support four key conclusions: first, in both BALB/c and C57Bl/6 mice, the majority of the antigen-experienced CD4 T cells in the lungs are Th1 cells at all time points tested following infection. Second, although Th17 cells were identified following infection, neither they nor Th2 cells were present in numbers significantly elevated above naïve at any time point post-infection, in either of the mouse strains tested. Third, the proportions of the CD4 T cell response after IAV infection belonging to Th1, Tfh, or Treg subsets is distinct in the two mouse strains: in C57Bl/6 mice, the Th1 and Tfh subsets represent stable proportions of the total CD4 T cell pool throughout infection, but only the Th1 and Treg contingent is stable throughout infection in BALB/c mice. Finally, in the lungs of C57Bl/6 mice, there is a much larger Tfh response than in BALB/c mice, whereas the opposite is true for the Treg response, which is larger in BALB/c mice. These four key findings show that the composition of the CD4 T cell response in the lungs after IAV varies over the course of infection, and has significant time-independent differences between C57BL/6 and BALB/c mice.

## Discussion

The work detailed herein offers new insights into the endogenous CD4 T cell response in the lungs of BALB/c and C57Bl/6 mice during infection with IAV. We demonstrate that while the endogenous NP_311_-specific CD4 T cell response is largely IFNγ-focused, with cells presumably belonging to the Th1 subset, NP_311_-specific CD4 T cells represent only a small fraction of the total antigen-experienced CD4 T cells in the lungs following infection. Further, our results suggest that the kinetics of their accumulation in the lungs is consistent with what has been previously reported for adoptively transferred CD4 T cells specific for a single antigen. Using the surrogate markers CD49d and CD11a, we show for the first time that the endogenous antigen-specific CD4 T cell response in the lungs is not only quite large, but also diverse, and that the kinetics of accumulation of antigen-experienced CD4 T cells in the lungs of BALB/c and C57Bl/6 mice is similar, but with several key differences in the relative abundance of the T cell subsets present. Most notably, although T-bet^+^ (Th1 polarized) CD4 T cells predominate in the lungs of both mouse strains, the second most abundant subset in BALB/c mice is Foxp3^+^ (Treg polarized) CD4 T cells, whereas in C57Bl/6 it is Bcl-6^+^ (Tfh polarized) CD4 T cells.

One important aspect of these studies is our validation of the use of the surrogate markers CD49d and CD11a for tracking antigen-specific CD4 T cell responses in the lungs during IAV infection. The surrogate marker approach is powerful in the context of IAV infection because the IAV-specific CD4 T cell repertoire recognizes a large number of antigens within IAV, each of which may only be recognized by a small number of cells (20). Further, our results argue that this surrogate marker approach is also well-suited to allow resolution of the T cell response under conditions where antigen specificity of the T cell response is not completely known, such as in outbred populations or infections with novel pathogens (29).

Our finding that Th1 cells are the most abundant population of antigen experienced CD4 T cells regardless of mouse strain is consistent with previous reports using adoptively-transferred memory cells (10). Perhaps, more interesting and surprising are the differences in the relative abundance on the remaining Th subsets in the lungs of C57Bl/6 compared with BALB/c mice. Differences in the immune response of different mouse strains and substrains to injuries, cancers and infections including IAV are well-appreciated (38), but this is the first time, to our knowledge, that the kinetics and subset distribution of the complete endogenous CD4 T cell response within the lungs during IAV has been directly compared in BALB/c and C57Bl/6 mice. It is anecdotally well-established that BALB/c mice are more susceptible to disease morbidity and mortality than otherwise identical C57BL/6 mice infected with the same dose of IAV. Interestingly, BALB/c mice have been reported to be more susceptible than C57Bl/6 mice to infection with the intracellular parasite *Leishmania major* due to an increased regulatory T cell response in BALB/c mice (18). While the presence of Treg during IAV infections has been shown to reduce pathology (34, 35), it is possible that the increased number and frequency of regulatory T cells we observed in IAV-infected BALB/c mice above the levels observed in C57BL/6 mice are suppressing some protective aspect of the immune response leading to increased disease severity.

In conclusion, the experiments described herein show that the CD4 T cell response to IAV in the lungs is robust and diverse, initially appearing in significant numbers at day 6–7 p.i., and peaking at day 10 p.i. in both BALB/c and C57Bl/6 mice. This study also supports the utility of using CD49d and CD11a for tracking antigen-experienced CD4 T cells during an acute infection, as 99% of cells specific for the NP_311−325_ epitope were also CD49d^+^CD11a^hi^. Finally, we show that the endogenous CD4 T cell response in the lungs during IAV is predominantly Th1 polarized, but has Treg and Tfh contingents in different proportions, dependent on host strain. Together these findings provide an important comparison of the kinetics and composition of the endogenous CD4 T cell response in the lungs in two widely used murine models of IAV infection.

## Ethics Statement

This study was carried out in accordance with the recommendations of NIH Guide for Care and Use of Laboratory Animals. The protocol was approved by the Institutional Animal Care and Use Committee of the University of Iowa.

## Author Contributions

KL conceived the research. EH, ZZ, and KL designed the experiments, analyzed data, interpreted the results, and wrote and edited the manuscript. EH and ZZ conducted the experiments. All authors reviewed and approved the manuscript.

### Conflict of Interest

The authors declare that the research was conducted in the absence of any commercial or financial relationships that could be construed as a potential conflict of interest.
