# Supplementary material for: Kinetics and Phenotype of the CD4 T Cell Response to Influenza Virus Infections
Source: Front Immunol. 2019 Oct 2;10:2351. doi: 10.3389/fimmu.2019.02351 (PMC6783515; doi:10.3389/fimmu.2019.02351)
Supplement: Supplementary file 1 [file Data_Sheet_1.PDF]

Supplementary Data Sheet 1

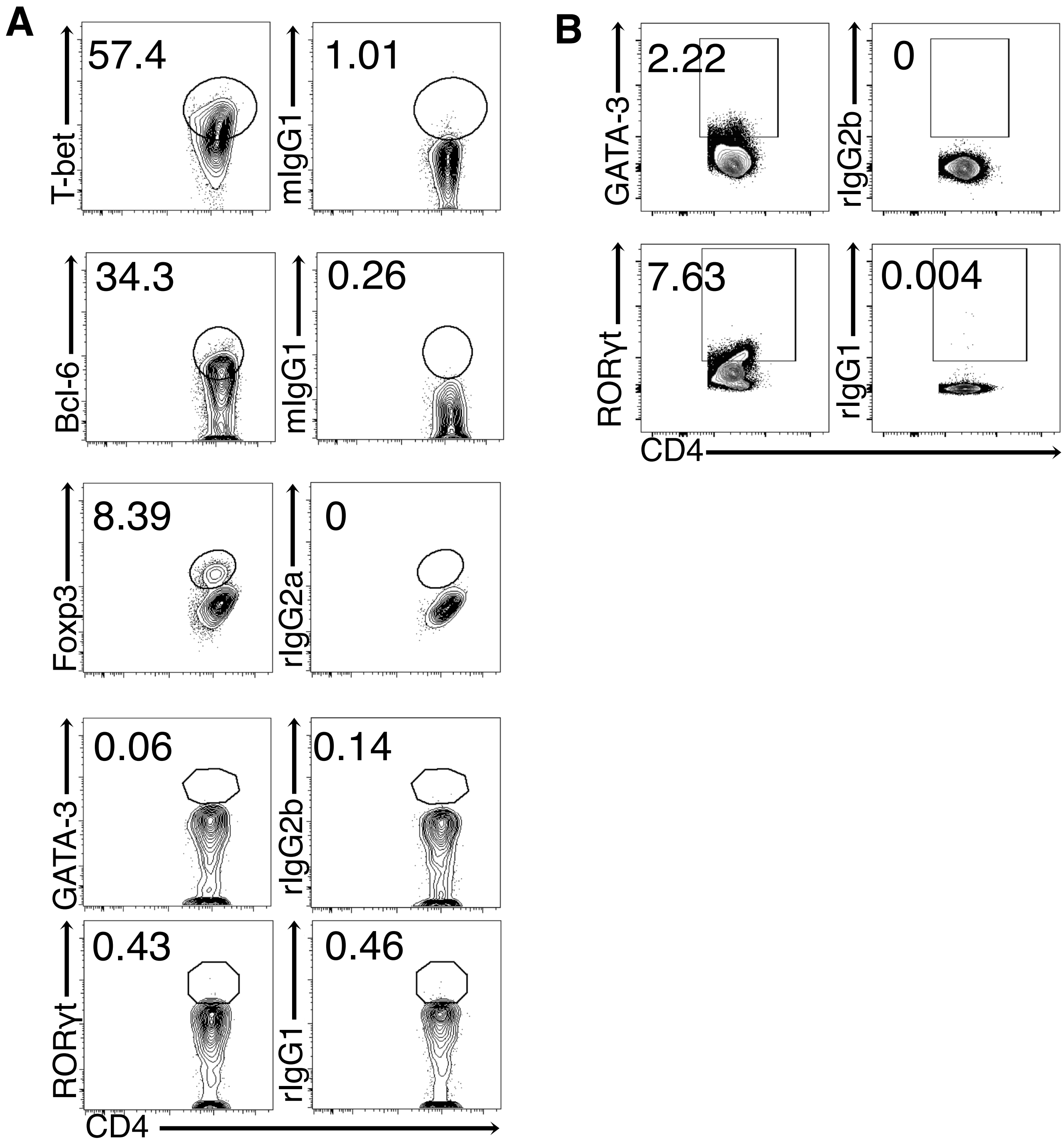

Supplementary Data Sheet 1. Representative staining for data shown in Figure 4. A) $CD4^+CD49d^+CD11a^{hi}$ lung cells from day 7 p.i. $0.05LD_{50}$ IAV infected C57BL/6 mice were stained with the indicated anti-transcription factor antibodies or isotype controls. B) As a positive control, thymocytes from a naïve C57BL/6 mouse were stained with the indicated anti-transcription factor antibodies or isotype controls.
